# Comparison of the Postoperative Analgesic Effects between Ultrasound-Guided Transmuscular Quadratus Lumborum Block and Thoracic Paravertebral Block in Laparoscopic Partial Nephrectomy Patients: A Randomized, Controlled, and Noninferiority Study

**DOI:** 10.1155/2023/8652596

**Published:** 2023-02-20

**Authors:** Jin Wang, Xulei Cui, Liying Ren, Xu Li, Yuelun Zhang, Yi Xie, Zhigang Ji, Yuguang Huang

**Affiliations:** ^1^Department of Anaesthesiology, Peking Union Medical College Hospital, Chinese Academy of Medical Sciences, Beijing, China; ^2^Central Research Laboratory, Peking Union Medical College Hospital, Chinese Academy of Medical Sciences, Beijing, China; ^3^Department of Urological Surgery, Peking Union Medical College Hospital, Chinese Academy of Medical Sciences, Beijing, China

## Abstract

**Background:**

This prospective, randomized, double-blinded, noninferiority study aimed to compare the effects of analgesia and recovery between transmuscular quadratus lumborum block (TMQLB) and paravertebral block (PVB).

**Methods:**

Sixty-eight, American Society of Anesthesiologists level I–III patients, who underwent laparoscopic partial nephrectomy in Peking Union Medical College Hospital were randomly allocated to either TMQLB or PVB group (independent variable) in a 1 : 1 ratio. The TMQLB and PVB groups received corresponding regional anesthesia preoperatively with 0.4 ml/kg of 0.5% ropivacaine and follow-up at postoperative 4, 12, 24, and 48 hours. The participants and outcome assessors were blinded to group allocation. We hypothesized that the primary outcome, postoperative 48-hour cumulative morphine consumption, in the TMQLB group was not more than 50% of that in the PVB group. Secondary outcomes including pain numerical rating scales (NRS) and postoperative recovery data were dependent variables.

**Results:**

Thirty patients in each group completed the study. The postoperative 48-hour cumulative morphine consumption was 10.60 ± 5.28 mg in the TMQLB group and 6.40 ± 3.40 mg in the PVB group. The ratio (TMQLB versus PVB) of postoperative 48-hour morphine consumption was 1.29 (95% CI: 1.13–1.48), indicating a noninferior analgesic effect of TMQLB to PVB. The sensory block range was wider in the TMQLB group than in the PVB group (difference 2 dermatomes, 95% CI 1 to 4 dermatomes, *P*=0.004). The intraoperative analgesic dose was higher in the TMQLB group than in the PVB group (difference 32 *µ*g, 95% CI: 3–62 *µ*g, *P*=0.03). The postoperative pain NRS at rest and on movement, incidences of side effects, anesthesia-related satisfaction, and quality of recovery scores were similar between the two groups (all *P*  >  0.05).

**Conclusions:**

The postoperative 48-hour analgesic effect of TMQLB was noninferior to that of PVB in laparoscopic partial nephrectomy. This trial is registered with NCT03975296.

## 1. Introduction

Ultrasound-guided transmuscular quadratus lumborum block (TMQLB), first proposed by Børglum et al. in 2013 [1], is an emerging fascial plane block that injects local anesthetics (LA) between the psoas major muscle (PM) and quadratus lumborum muscle (QLM) [2, 3].

It has been speculated that TMQLB might produce an analgesic effect similar to that of the thoracic paravertebral block (TPVB), because a previous study on cadavers showed that the injectate injected with the TMQLB approach could spread via a pathway posterior to the arcuate ligaments and into the thoracic paravertebral space, thus infiltrating the thoracic somatic nerves and sympathetic trunk to provide both somatic and visceral analgesia [4]. A retrospective study conducted by Lee et al. [5] on patients receiving radical cystectomy also showed that the postoperative day 0 opioid consumption and pain scores were similar between patients receiving continuous TPVB and single-shot TMQLB. A single-shot nerve block is superior to a continuous nerve block in reducing block-related complications [6] and facilitating patient movement and nursing.

To the best of our knowledge, no prospective clinical trials have compared the analgesic effects of single-shot TMQLB and TPVB. Both TMQLB and TPVB have been reported to be effective analgesic modalities for laparoscopic urological surgeries [7–12]. Compared to TPVB, TMQLB avoids the risk of pneumothorax and has lower coagulation requirements. Therefore, we aim to compare the effects of perioperative analgesia and recovery quality between TMQLB and TPVB in patients undergoing laparoscopic partial nephrectomy. We hypothesized that the analgesic effect of preoperative single-shot TMQLB is noninferior to that of TPVB in terms of postoperative 48-hour cumulative morphine consumption. The study results may help clinicians further understand the analgesic effect and mechanism of TMQLB and improve perioperative care.

## 2. Materials and Methods

### 2.1. Trial Design

This is a prospective, randomized, double-blind, noninferiority study. The study design was approved by the Institutional Review Board of Peking Union Medical College Hospital in Beijing, China (ZS-1559), and was registered at ClinicalTrials.gov (https://clinicaltrials.govNCT03975296) on June 3, 2019. Patient enrollment was started on June 10, 2019. Written informed consent was obtained from all participants before participating. This manuscript adheres to the applicable Consolidated Standards of Reporting Trials guidelines, and the study was conducted in accordance with the Declaration of Helsinki.

### 2.2. Participants

The inclusion criteria were as follows: (1) 18–70 years old; (2) American Society of Anesthesiologists level I–III; and (3) scheduled for laparoscopic partial nephrectomy. The exclusion criteria were as follows: (1) allergy to medications used; (2) coagulopathy or use of anticoagulants; (3) history of substance abuse; (4) inability to describe the pain (e.g., neuropsychiatric disorder, language barrier); and (5) participation in other clinical trials.

### 2.3. Interventions

All blocks were performed in a dedicated procedure room before surgery. After applying standard monitors, supplemental oxygen, and intravenous access, patients were placed in the lateral decubitus position with the operation side upwards.

**Figure 1:**
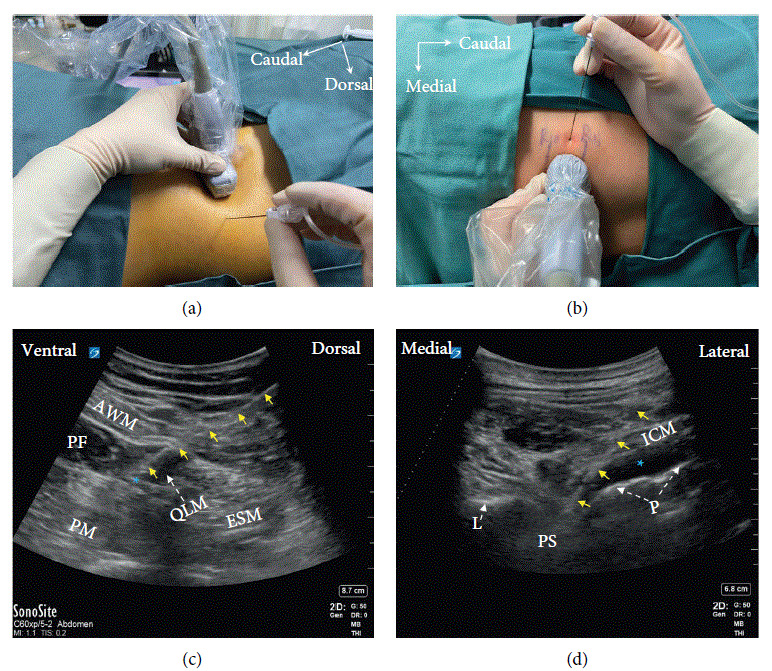
Ultrasound-guided transmuscular quadratus lumborum block (a, b) and thoracic paravertebral block (c, d). AWM: abdominal wall muscle, EPF: extraperitoneal fat, ESM: erector spine muscle, PM: psoas muscle, QLM: quadratus lumborum muscle, P: pleura, PS: paravertebral space, L: lamina, ICM: intercostal muscle, ^*∗*^: LA, yellow arrows: needle.

In the TMQLB group, TMQLB was performed using the approach described by Børglum et al. [1]. A curved array transducer (Sonosite X-Port, USA) was placed on the transverse plane in the abdominal flank caudal to the rib. The transducer was then moved dorsally, keeping the transverse orientation until the “shamrock sign” appeared. A 22-G needle (Paujunk) was inserted in the plane with the tip advanced from the dorsal to the ventral direction, penetrating the ventral fascia of the QLM. After the correct needle tip position was confirmed with hydrodissection, 0.4 ml/kg 0.5% ropivacaine was injected between the QLM and PM (Figures 1(a) and 1(c)).

In the TPVB group, TPVB was performed using the approach described by Renes et al. [13]. A curved array transducer (Sonosite X-Port, USA) was placed on the paramedian sagittal plane to identify the T10 intercostal space. Then, the transducer was rotated to align with the intercostal space and adjusted to identify the paravertebral space between the sliding pleura and inner intercostal membrane. A 22-G needle (Paujunk) was inserted in the plane and advanced from the lateral to the medial direction until the needle tip reached the paravertebral space. After the correct needle tip position was confirmed with hydrodissection, 0.4 ml/kg 0.5% ropivacaine was injected into the space (Figures 1(b) and 1(d)), producing a pleural depression sign.

Sensory block dermatomes were tested 30 minutes after the nerve block. A cold stimulus was applied along the midclavicular line to compare bilateral sensory changes. A cold sensation regression compared to the contralateral side of the same dermatome was considered an effective block. The total numbers and levels of sensory block dermatomes were recorded and compared between the two groups. Heart rate and blood pressure were also recorded as preoperative vital signs. Block-related complications, including hemorrhage, organ injury, pneumothorax (related to TPVB), LA toxicity, and injection site infection, were also recorded.

All patients underwent standardized general anesthesia with tracheal intubation. Anesthesia was induced with 2 mg/kg propofol, 1 *µ*g/kg fentanyl, and 0.8 mg/kg rocuronium and maintained with sevoflurane in an air-oxygen mixture and a BIS value of 40 to 60. The baseline heart rate and blood pressure were defined as the mean value of the heart rate and blood pressure measured on the three consecutive days before surgery. The intraoperative heart rate was maintained within ±10 bpm of the baseline and recorded every ten minutes. The intraoperative blood pressure was maintained within ±20% of the baseline and recorded every ten minutes. Fentanyl was administered as needed with 1 *µ*g/kg per bolus. Intraoperative medications and fluids were recorded by the anaesthesiologists during the surgery. Sevoflurane was stopped, and neostigmine was given after the innermost layer of the wound was closed.

After surgery, both groups received electronic patient-controlled intravenous analgesia (PCIA) pumps (Apon Medical Corp. China). Morphine was given by pressing the self-dosing button when the patients needed it, with the parameters set at 1.5–2 mg per bolus at a 5-minute lockout interval and an upper limit of 6–8 mg per hour, without continuous background infusion. Parecoxib, as a rescue for insufficient analgesia, was administered if the patient still complained of pain NRS ≥4 after using PCIA. Postoperative follow-up and outcome assessment was performed by an experienced anesthesiologist who was blinded to the group allocation.

### 2.4. Outcomes

The primary outcome was postoperative 48-hour cumulative morphine consumption recorded by the electronic PCIA pump. The secondary outcomes included: (1) sensory block dermatomes 30 min after blockade; (2) intraoperative hemodynamic changes and medications used; (3) NRS pain scores at rest and during movement at postoperative 0, 4, 12, 24 and 48 hours; (4) cumulative morphine consumption at postoperative 4, 12 and 24 hours; (5) postoperative recovery data, including gas passing, urination and off-bed times, and incidences of postoperative nausea and vomiting (PONV), pruritus and dyspnea; (6) anesthesia-related satisfaction scores evaluated by a 1- to 5-point Likert scale, with 1 point defined as very unsatisfied, 2 points defined as unsatisfied, 3 points defined as acceptable, 4 points defined as satisfied, and 5 points defined as very satisfied [14, 15]; (7) quality of recovery evaluated by the self-assessment15-item quality of recovery scale [16, 17]; and (8) length of hospital stay.

### 2.5. Sample Size

The sample size was calculated using the noninferiority module of PASS 11 based on the primary endpoint according to the noninferiority hypothesis. According to a previous study, the predetermined noninferiority limit (*δ*) was set to a 50% increase over 48 hours of cumulative morphine consumption [18]. Based on a preliminary analysis of 20 patients (unpublished), a mean value of 6.5 mg and a standard deviation (SD) of 4.4 mg were assumed for the morphine consumption distribution. With a significance level of 0.05 and a power of 80%, 30 patients were required in each group. Assuming a 10% dropout rate, we decided to enroll 34 patients per group.

### 2.6. Randomization and Allocation

Patient enrollment, randomization, and allocation were performed by research team members. Patients were allocated to either the TMQLB or the PVB group at a 1 : 1 ratio based on computer-generated randomization results. Random numbers were concealed in a sequentially numbered opaque envelope, which was only opened by the block practitioner before block performance.

### 2.7. Blinding

All blocks were performed by a single experienced regional anesthesiologist (X.C.), together with a single assistant nurse who was otherwise not involved in the study. All other research personnel and outcome assessors were blinded to group allocations. Patient blinding was maintained as much as possible by standardizing some of the perceptible block elements, such as positioning and LA injection volumes. However, differences in ultrasound probe placement and needle insertion site still existed.

### 2.8. Statistical Analysis

After testing the normality of the data distribution using the Q-Q plot, continuous variables with normal distribution, including postoperative cumulative intravenous morphine consumption, pain NRS score, heart rate, blood pressure, sevoflurane and fentanyl doses, urine output and crystalloid infusion volumes, recovery time, quality of life, and anesthesia-related satisfaction scores, were presented as the means ± SDs. Continuous variables with non-normal distribution, including intraoperative vasoactive agent doses, colloid, and blood product infusion volumes, were presented as medians and interquartile ranges. Categorical variables, including incidences of postoperative nausea, vomiting, pruritus, and dyspnea, were presented as numbers and percentages. The primary outcome, postoperative 48-hour cumulative morphine consumption (*Y*), was tested with a *Y*_TMQLB_/*Y*_TPVB_ ratio of less than 1.5 as null. Linear regression was performed on Ln(*Y*)∼group (TMQLB = 1, TPVB = 0) to obtain a coefficient B. Based on the formula Ln (*Y*_TMQLB_) − Ln (*Y*_TPVB_) = Ln (*Y*_TMQLB_/*Y*_TPVB_) = (*B∗* 1 + constant) − (B*∗*0 + constant) = *B*, *Y*_TMQLB_/*Y*_TPVB_ was obtained as *e*^*B*^. Noninferiority was established if the upper limit of the 95% CI of *e*^*B*^ < 1.5. Postoperative intravenous morphine consumption and pain NRS scores were also compared between the TMQLB and TPVB groups using two-wayrepeated-measures ANOVA. No post hoc tests were performed because there were only two treatment groups. Other outcomes were compared between the TMQLB and TPVB groups using the independent *t-test* or the Mann–Whitney test for continuous variables and the chi-square test or Fisher's exact test for categorical variables. The group allocation was an independent variable and all outcomes were dependent variables. Data analysis was performed using SPSS software (version 25.0, SPSS Inc., USA). For all analyses, *P* <  0.05 was considered to indicate significance.

## 3. Results

### 3.1. Study Flow and Baseline Characteristics

**Figure 2:**
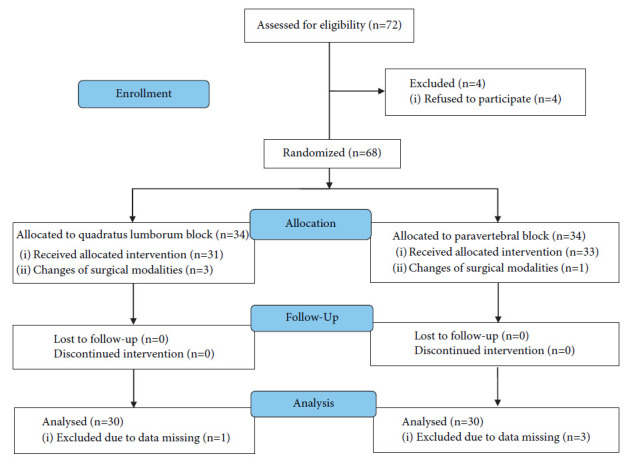
Consolidated standards of reporting trials flow diagram showing patient progress through the study phases.

From Jun 29^th^_,_ 2019 to Jan11^th^_,_ 2021, a total of 72 patients scheduled for laparoscopic partial nephrectomy were assessed for eligibility. Four patients who refused to participate were excluded. All enrolled patients (*n* = 68) were randomly assigned to one of the two treatment groups (*n* = 34 each). Eight patients were excluded due to changes in surgical modality (*n* = 4) and missing data (*n* = 4). Thirty patients in each group were included in the final analysis (Figure 2).

**Figure 3:**
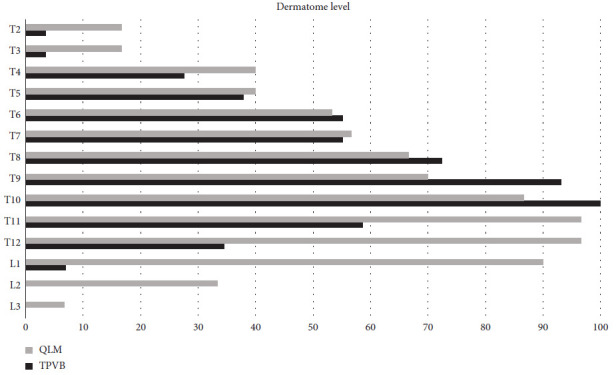
Range of dermatomal sensory block levels.

The baseline data are listed in Table 1. The mean age, sex ratio, body mass index, preoperative pain NRS, surgical side, and surgical time were similar between the two groups (all *P*  >  0.05). The sensory block dermatomes are shown in Table 2 and Figure 3. The sensory block ranges of TMQLB and TPVB were from the T2 to L3 levels and from the T2 to L1 levels, respectively. The average sensory block range of TMQLB was wider than that of TPVB (difference 2 dermatomes; 95% CI: 1 to 4 dermatomes; *P*=0.004).

### 3.2. Postoperative Analgesia

**Figure 4:**
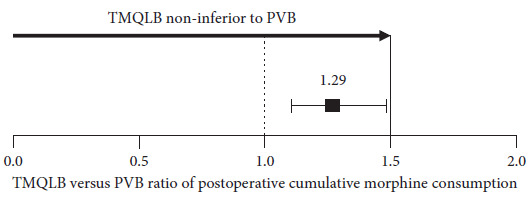
Noninferiority diagram of postoperative 48-hour cumulative morphine consumption differences in TMQLB and TPVB group. Error bar: 95% confidence interval.

The postoperative analgesia data are listed in Table 2 and Figure 4. The postoperative 48-hour cumulative morphine consumption was 6.40 ± 3.40 mg in the TPVB group and 10.60 ± 5.28 mg in the TMQLB group. The mean difference in postoperative 48-hour cumulative morphine consumption between the TMQLB and TPVB groups was 4.20 mg (95% CI 1.90 to 6.50 mg), with a *P* value of 0.001. The ratio (TMQLB versus TPVB) of morphine consumption at 48 hours postoperatively was 1.29 (95% CI 1.13–1.48). Because the upper limit of the 95% CI was lower than the prespecified noninferiority margin, noninferiority was established. The analgesic effect of TMQLB was noninferior to that of TPVB 48 hours after laparoscopic partial nephrectomy. The mean differences in postoperative 12-hour and 24-hour cumulative morphine consumption between the TMQLB and TPVB groups were 2.09 mg (95% CI 0.38 to 3.79 mg, *P*=0.017) and 3.61 mg (95% CI 1.49 to 5.73 mg, *P*=0.001), respectively. Pain NRS both at rest and during movement at postoperative 0, 4, 12, 24, and 48 hours were similar between the two groups (all *P*  >  0.05). The rescue analgesic rate was similar between the two groups (*P*  >  0.05).

### 3.3. Intraoperative and Postoperative Recovery Data

The intraoperative hemodynamic changes and medications used are listed in Table 3. The baseline heart rate and blood pressure were similar between the two groups (all *P*  >  0.05). After nerve block but before general anesthesia, the preoperative diastolic blood pressure (difference 7 mmHg; 95% CI: 1 mm Hg to 14 mm Hg; *P* = 0.029) was lower in the TPVB group than in the TMQLB group. The intraoperative heart rate and blood pressure were similar between the two groups (all *P*  >  0.05). The TMQLB group used more sevoflurane (difference 0.15%, 95%CI 0.02% to 0.27%, *P*=0.022) and fentanyl (difference 32 µg, 95%CI 3 µg to 62 µg, *P*=0.031) but less ephedrine (difference 6 mg, 95%CI 0 mg to 6 mg, *P*=0.022) than the TPVB group. The fluid input and output volumes were similar between the two groups (all *P*  >  0.05).

The postoperative recovery data are listed in Table 4. The gas passing, urination, and off-bed times were similar between the two groups (all *P*  >  0.05). The incidences of PONV, pruritus, and dyspnoea were similar between the two groups (all *P*  >  0.05). The anesthesia-related satisfaction scores evaluated 48 hours postoperatively and 15-item quality of recovery scores evaluated 3 and 5 days postoperatively were similar between the two groups (all *P*  >  0.05).

No regional block-related adverse events were reported.

## 4. Discussion

To the best of our knowledge, this study was the first prospective, randomized, controlled study comparing perioperative analgesia and recovery between TMQLB and TPVB in patients undergoing laparoscopic partial nephrectomy. The results demonstrated that the analgesic effect of TMQLB was noninferior to that of TPVB in terms of postoperative 48-hour cumulative morphine consumption. Both groups achieved good postoperative analgesia with similar pain NRS scores at rest and during movement. No significant differences in postoperative recovery data between the two groups were noted.

The perioperative pain due to laparoscopic nephrectomy consists of both somatic and visceral elements. The somatic pain caused by gas distention of the abdominal wall, abdominal port placement, and dissection of the abdominal cavity arises from the spinal nerves of T6 to T12. The visceral pain in the renal pelvis is innervated by the sympathetic trunk from T12 to L2 [19]. In this study, with 0.4 ml/kg ropivacaine, TMQLB blocked dermatomes from T2 to L3, similar to Zhu et al.'s study results [20], and TPVB blocked an average of 5 dermatomes, also similar to previous studies' results [21, 22]. Both TMQLB's and TPVB's sensory block ranges covered the nerve distribution area responsible for pain conduction of laparoscopic partial nephrectomy, which could explain the noninferior pain relief effect of TMQLB compared with TPVB in the current study.

When injected with the same dosages of ropivacaine, TMQLB produced a wider sensory block range than TPVB. This phenomenon might be explained by the different LA spreading patterns of these two techniques. TMQLB injects LA within a latent fascial space between the PM and QLM. The fascial planes follow the PM and QLM cranially through the medial and lateral arcuate ligaments and the aortic hiatus of the diaphragm, forming the endothoracic fascia and inferior diaphragmatic fascia [2, 4]. Hence, LA can spread cranially along the endothoracic fascia and infiltrate the ventral ramus of the thoracic spinal nerve and generate a sensory block effect sufficient to cover the somatic pain caused by laparoscopic partial nephrectomy. The endothoracic fascia is a loose, mesh-like connective tissue of approximately 250-µm thickness [23], and LA injected via TMQLB can spread along it easily, generating a wide sensory block range. However, the thoracic paravertebral space is wedge-shaped with solution-containing capacity. Thus, LA injected via TPVB tends to spread limitedly and results in a relatively aggregate blocking pattern.

Despite a noninferior postoperative 48-hour analgesic effect of TMQLB compared to TPVB based on an *H*_1_/*H*_0_ < 1.5 hypothesis in our study, we did note significant statistical differences in intraoperative and postoperative 24-hour and 48-hour analgesic doses between the TMQLB and TPVB groups based on an *H*_1_ = 0 hypothesis. This outcome can be explained statistically and might also imply a potential inferior analgesic effect, especially the visceral analgesic effect of TMQLB compared to TPVB due to differences in the sympathetic block effect. The lower postblock diastolic blood pressure of TPVB compared to TMQLB also supports this inference. The sympathetic block effect of TPVB can be explained mainly by the following three mechanisms: (1) injection of LA directly anterior to the endothoracic fascia and into the extrapleural compartment [24], where the sympathetic trunk lies [13, 25]; (2) spreading of LA from the subendothoracic compartment into the extrapleural compartment through fenestrations of the endothoracic fascia; and (3) diffusion of LA into the epidural space, especially when the injection is medial. However, the sympathetic block effect of TMQLB remains controversial. While Børglum et al.'s study of sixteen cadavers showed that all dye injected with TMQLB could spread into the paravertebral space and infiltrate the sympathetic trunk, Gadsden et al.'s study of one cadaver showed that the transverse oblique paramedian approach QLB at L3 completely spared the paravertebral space [4, 26]. A healthy volunteer study on other QLB approaches also showed inconsistent results [27]. Hence, the sympathetic block, as well as the visceral analgesic effect, with TMQLB was considered not as reliable as that with TPVB.

For postoperative recovery, the incidences of adverse events, patients' satisfaction with anesthesia scores, and 15-item quality of recovery scores were all similar between the two groups, indicating a comparable safety and recovery profile of both blocks.

There are several noteworthy limitations of the current study. First, the ultrasound transducer placement and needle injection sites were different between the TMQLB and TPVB groups; thus, patients might not be fully blinded to the injection methods. However, the chances of identifying different block techniques were low in patients without medical backgrounds. Second, this study did not provide information about differences in pharmacodynamics and risks of systemic toxicity between the two blocks since we did not measure serum LA concentrations. To minimize the risk of toxicity, further studies are needed to determine the minimum dosage of LA that produces the maximum beneficial clinical effects. Third, this study was a single-center study in a specific surgical setting and suffered from all of the shortcomings of this type of study. Since laparoscopic nephrectomy is a moderately invasive surgery with a less invasive wound than open surgery [28–30] the pain produced by it might not be sufficiently strong to distinguish the difference in analgesia between TMQLB and TPVB. Further studies are required to investigate the analgesic effects of TMQLB in other types of surgery and patient populations.

## 5. Conclusions

The present study demonstrated a noninferior postoperative analgesic effect of TMQLB compared to T10 level TPVB in terms of postoperative 48 hours cumulative morphine consumption in laparoscopic partial nephrectomy. TMQLB could be considered a substitution for TPVB in specific surgical settings and patient populations, and it is worthy of further investigation.

## Figures and Tables

**Table 1:** Patient characteristics.

Parameter	TMQLB group	TPVB group
Sample size, *n*	30	30
Mean age (SD) in years	50 (10)	53 (12)
Male, *n* (%)	19 (63.3%)	10 (33.3%)
Mean body mass index (SD) in kg·m^−2^	26.11 (3.73)	24.98 (3.20)
Mean pre-op NRS (SD) in points	0.10 (0.55)	0.13 (0.43)
Right-side surgery, *n* (%)	18 (60.0%)	16 (53.3%)
Mean duration of surgery (SD) in minutes	106 (39)	117 (33)

TMQLB: transmuscular quadratus lumborum block, TPVB: thoracic paravertebral block, Op: operation, NRS: numerical rating scale.

**Table 2:** Postoperative cumulative morphine consumption and pain NRS of transmuscular quadratus lumborum block and paravertebral block at different time points.

	TMQLB group	TPVB group	Difference (95% CI)	*P* value
Sample size, *n*	30	30	—	—
Mean postoperative cumulative morphine consumption (SD) in mg				
48-hour	10.60 (5.28)	6.40 (3.40)	4.20 (1.90, 6.50)	0.001^*∗*^
4-hour	1.64 (1.88)	1.00 (1.41)	0.64 (−0.22, 1.50)	0.139
12-hour	4.75 (4.03)	2.67 (2.27)	2.09 (0.38, 3.79)	0.017^*∗*^
24-hour	7.69 (5.07)	4.08 (2.73)	3.61 (1.49, 5.73)	0.001^*∗*^
Pain NRS at rest (SD) in points				
0-hour	0.73 (1.05)	0.53 (1.07)	0.20 (−0.35, 0.75)	0.468
4-hour	1.53 (1.25)	1.52 (1.51)	0.02 (−0.70, 0.73)	0.963
12-hour	2.33 (1.79)	2.45 (1.50)	−0.12 (−0.97, 0.74)	0.785
24-hour	1.73 (1.48)	1.53 (1.27)	0.20 (−0.51, 0.91)	0.577
48-hour	1.03 (1.10)	0.92 (1.07)	0.12 (−0.44, 0.68)	0.678
Pain NRS on movement (SD) in points				
0-hour	1.30 (1.58)	0.63 (0.93)	0.67 (0.00, 1.34)	0.051
4-hour	3.07 (1.46)	2.72 (1.53)	0.35 (−0.42, 1.12)	0.368
12-hour	3.70 (1.74)	3.63 (1.03)	0.07 (−0.67, 0.81)	0.857
24-hour	3.33 (1.56)	3.30 (1.00)	0.03 (−0.64, 0.71)	0.922
48-hour	2.53 (1.41)	2.42 (1.11)	0.12 (−0.54, 0.77)	0.723
Mean sensory block dermatomes (SD) in segments				
Segments	8 (4)	5 (3)	2 (1, 4)	0.004^*∗*^
Rescue rate, *n* (%)	4 (13.3%)	4 (13.3%)	1.00 (0.23, 4.43)	1.000

TMQLB: transmuscular quadratus lumborum block, TPVB: thoracic paravertebral block, NRS: numerical rating scale, OR: odds ratio, CI: confidence interval, SD: standard deviation. ^*∗*^Significant statistical difference.

**Table 3:** Intraoperative hemodynamic changes and medication used in transmuscular quadratus lumborum block and paravertebral block groups.

	TMQLB group	TPVB group	Difference (95% CI)	*P* value
Sample size, *n*	30	30	—	—
Mean heart rate in (SD) beats per minute				
Baseline	76 (7)	73 (8)	3 (−1, 7)	0.148
Pre-op	78 (14)	71 (10)	6 (0, 13)	0.055
Intra-op	66 (10)	64 (7)	2 (−3, 6)	0.464
Mean systolic blood pressure (SD) in mmHg				
Baseline	129 (14)	127 (12)	3 (−4, 9)	0.423
Pre-op	140 (23)	133 (21)	7 (−4, 19)	0.189
Intra-op	111 (9)	111 (15)	3 (−2, 9)	0.210
Mean diastolic blood pressure (SD) in mmHg				
Baseline	78 (9)	78 (6)	0 (−4, 4)	0.947
Pre-op	87 (14)	80 (10)	7 (1, 14)	0.029^*∗*^
Intra-op	77 (10)	74 (9)	4 (−2, 8)	0.195
Medications and fluids				
Mean sevoflurane (SD) in %	1.2 (0.2)	1.1 (0.2)	0.15 (0.02, 0.27)	0.022^*∗*^
Mean fentanyl (SD) in *μ*g	250 (66)	218 (44)	32 (3, 62)	0.031^*∗*^
Median urapidil (quartiles) in mg	0 (0, 0, 0, 10)	0 (0, 0, 0, 0)	0 (0, 0)	0.317
Median ephedrine (quartiles) in mg	1.5 (0, 0, 6, 18)	6 (0, 4.5, 12, 30)	6 (0, 6)	0.022^*∗*^
Median phenylephrine (quartiles) in mg	0 (0, 0, 0, 0.16)	0 (0, 0, 0, 0.6)	0 (0, 0)	0.644
Median atropine (quartiles) in mg	0 (0, 0, 0, 0.3)	0 (0, 0, 0, 0.3)	0 (0, 0)	0.570
Mean crystalloid (SD) in ml	1441 (325)	1365 (361)	76 (103, −256)	0.397
Median colloid (quartiles) in ml	0 (0, 0, 0, 500)	0 (0, 0, 0, 1000)	0 (0, 0)	0.621
Median RBC (quartiles) in U	0 (0, 0, 0, 0)	0 (0, 0, 0, 0)	0 (0, 0)	1.000
Median plasma (quartiles) in ml	0 (0, 0, 0, 0)	0 (0, 0, 0, 0)	0 (0, 0)	1.000
Median hemorrhage (quartiles) in ml	50 (0, 48, 50, 400)	50 (0, 20, 100, 700)	0 (0, 10)	0.856
Mean urine (SD) in ml	331 (249)	388 (260)	−58 (−189, 74)	0.383

TMQLB: transmuscular quadratus lumborum block, TPVB: thoracic paravertebral block, SBP: systolic blood pressure, DBP: diastolic blood pressure, MBP: mean blood pressure, op: operation, RBC: red blood cell, SD: standard deviation. ^*∗*^Significant statistical difference.

**Table 4:** Postoperative recovery data of transmuscular quadratus lumborum block and paravertebral block group.

Outcomes	TMQLB group	TPVB group	Difference/OR (95% CI)	*P* value
Sample size, *n*	30	30	—	—
Mean time (SD) in hours/days				
Gas passing (hours)	36 (17)	38 (17)	−2 (−11, 7)	0.631
Urination (hours)	58 (21)	57 (15)	1 (−8, 10)	0.823
Off-bed (hours)	53 (18)	50 (14)	3 (−6, 11)	0.524
Length of stay (days)	6 (1)	6 (2)	0 (−1, 1)	0.613
Incidence, *n* (%)				
Nausea	10 (33.3%)	8 (26.7%)	1.38 (0.45, 4.17)	0.573
Vomiting	10 (33.3%)	8 (26.7%)	1.38 (0.45, 4.17)	0.573
Pruritus	0 (0.0%)	1 (3.3%)	0.94 (0.06, 15.66)	0.963
Dyspnea	3 (10.0%)	3 (10.0%)	1.00 (0.19, 5.40)	1.000
Mean quality of recovery score (SD) in points				
Post-op day 3	127 (12)	124 (16)	4 (−4, 11)	0.314
Post-op day 5	139 (10)	138 (13)	1 (−5, 7)	0.765
Anesthesia related satisfaction (SD) in points				
Pre-op information	4.50 (0.51)	4.60 (0.56)	−0.10 (−0.38, 0.18)	0.473
Emergence	4.57 (0.63)	4.67 (0.48)	−0.10 (−0.39, 0.19)	0.490
Post-op analgesia	4.37 (0.62)	4.63 (0.49)	−0.27 (−0.55, 0.02)	0.068
PONV treatment	4.40 (0.72)	4.13 (0.90)	0.27 (−0.16, 0.69)	0.211
Anesthesia care	4.87 (0.90)	4.83 (0.38)	0.02 (−0.33, 0.39)	0.852

TMQLB: transmuscular quadratus lumborum block, TPVB: thoracic paravertebral block, PONV: postoperative nausea and vomiting, CI: confidence interval, SD: standard deviation.

## Data Availability

The raw data used to support the findings of this study are available from the corresponding author upon request.

## Supplementary Materials

Supplementary Materials
*Supplement 1.* CONSORT 2010 Checklist. *Supplement 2*. CONSORT Extension for Noninferiority and Equivalence Trials Checklist 2006. *Supplement 3*. Abstracts Checklist. The raw data is provided in Supplementary Materials.
