# Supplementary material for: Comparison of the Postoperative Analgesic Effects between Ultrasound-Guided Transmuscular Quadratus Lumborum Block and Thoracic Paravertebral Block in Laparoscopic Partial Nephrectomy Patients: A Randomized, Controlled, and Noninferiority Study
Source: Pain Res Manag. 2023 Feb 20;2023:8652596. doi: 10.1155/2023/8652596 (PMC9988391; doi:10.1155/2023/8652596)
Supplement: Supplementary Materials — Supplement 1. CONSORT 2010 Checklist. Supplement 2. CONSORT Extension for Noninferiority and Equivalence Trials Checklist 2006. Supplement 3. Abstracts Checklist. The raw data is provided in Supplementary Materials. [file 8652596.f1.zip › raw data.pdf]

序号	sex	age	BMI	Side	Group	HR基础	HR入室	HR平均	SBP基础	SBP入室
1	2	55	23.36	1	2	78	62	63	122	120
2	2	40	24.84	1	2	82	82	80	109	108
3	2	52	28.65	1	2	61	58	55	125	122
4	1	44	20.07	1	2	76	74	56	128	132
5	1	56	27.10	2	2	65	67	65	122	138
7	2	70	20.90	1	2	80	70	70.3	116	119
12	1	47	28.71	2	2	70	77	63	148	165
13	2	58	23.05	1	2	64	61	52	129	126
15	2	59	25.39	2	2	66	61	59	135	168
16	2	28	19.07	2	2	90	84	61	106	124
17	1	51	29.35	1	2	79	71	64	138	124
18	2	62	26.13	2	2	70	84	65	120	124
20	1	61	27.47	2	2	72	78	68	147	177
25	2	49	25.35	1	2	78	80	69	132	144
29	1	47	25.83	2	2	80	73	66	111	128
30	1	64	28.41	2	2	80	69	82	133	143
32	2	45	22.89	1	2	66	55	55	117	119
38	1	56	23.18	1	2	76	77	61	122	121
39	2	17	26.08	2	2	82	74	70	125	112
40	2	50	23.24	2	2	68	59	64	129	142
42	2	61	27.01	1	2	76	76	64	150	76
43	2	53	22.27	1	2	60	44	54	121	59
44	2	56	33.20	1	2	75	80	59	142	140
45	1	68	25.51	2	2	80	86	65	140	150
46	2	46	24.39	1	2	65	78	78	109	112
48	2	60	20.55	2	2	68	65	71	113	162
51	2	74	22.77	1	2	78	88	63	127	151
54	1	60	22.32	2	2	62	63	53	127	151
58	2	53	23.14	2	2	65	70	65	120	119
60	2	56	29.22	1	2	72	78	65	138	128
6	1	67	19.59	1	4	59	54	55	155	178
8	1	36	25.39	2	4	79	87	76	120	137
9	2	61	28.04	2	4	75	78	60	120	129
10	1	53	29.07	2	4	69	63	56	115	141
11	1	56	30.04	2	4	80	90	73	132	158
14	1	53	21.34	2	4	78	90	74	97	102
19	2	54	20.55	2	4	62	51	52	133	120
21	2	54	25.96	2	4	81	81	62.4	122	135
22	1	43	25.71	1	4	70	81	67.92	123	125
23	1	26	27.76	1	4	80	73	62	137	126
24	2	65	28.76	1	4	79	90	79.3	142	145
26	2	40	22.03	2	4	85	116	92.5	118	137
27	1	49	25.95	1	4	78	73	80.25	124	123
28	1	45	23.88	1	4	88	114	78	123	140
31	1	62	25.93	1	4	65	63	52	112	122
33	1	39	30.39	2	4	78	66	60	133	119
34	1	62	25.47	1	4	71	79	58	134	192
35	2	52	23.50	1	4	78	76	62	139	166

36	2	53	20.70	2	4	77	80	64	135	175
37	2	29	19.57	1	4	78	79	68	117	127
41	1	61	28.09	1	4	68	67	57	123	133
47	1	44	29.04	1	4	85	79	73.4	137	144
49	1	52	26.42	2	4	68	81	53	151	180
50	1	58	32.08	1	4	73	66	57	122	106
52	1	37	29.59	1	4	72	75	67	128	157
53	2	52	23.73	2	4	71	74	56	133	135
55	2	47	33.30	1	4	87	90	67	170	168
56	1	44	27.55	1	4	80	65	67	126	112
57	1	48	30.67	1	4	71	67	62	137	121
59	2	64	23.23	1	4	83	86	85	124	154

SBP平均	DBP基础	DBP入室	DBP平均	sev平均	手术时间分钟	芬太尼ug	亚宁定mg
100	69	78	74	1	80	226	0
101	66	63	60	0.93	65	240	0
108	77	74	65	1.06	133	250	0
114	83	84	79	1.26	80	160	0
105	73	93	76	59	192	59	0
112	79	91	87	59	125	160	0
112	82	97	79	59	94	200	0
117	80	68	76	1	66	160	0
100	85	89	60	1	82	260	0
106	82	87	81	1.07	129	188	0
100	75	85	73	1.43	108	270	0
109	82	66	76	1.12	100	284	0
109	83	68	67	0.95	125	260	0
109	75	89	79	1.28	112	250	0
114	75	86	82	59	100	180	0
115	80	79	77	59	144	230	0
113	71	75	73	59	80	165	0
104	74	80	64	59	96	230	0
92	82	90	57	1.46	17	210	0
125	74	74	82	0.9	96	180	0
119	90	58	81	1.16	107	170	0
112	76	59	79	59	127	120	0
124	80	80	77	0.92	83	250	0
122	90	85	86	59	144	200	0
113	65	62	62	59	57	200	0
114	73	80	70	1.07	90	274	0
106	78	74	56	0.96	71	220	0
138	77	92	89	1.05	114	240	0
102	70	78	79	1.13	202	295	0
111	82	87	79	1.2	156	240	0
151	79	81	84	0.9	105	240	10
124	87	83	86	1.67	157	165	0
128	79	80	81	1.2	183	340	0
111	74	100	88	1.06	150	250	0
110	88	110	82	1.1	90	290	0
110	65	102	89	1.19	120	280	0
119	68	64	81	1.05	90	180	0
132	80	84	90.9	1.7	98	175	0
106.28	74	97	81.57	1.22	133	250	0
109	74	72	60	1.32	132	250	0
108.4	88	69	62	1.02	150	150	0
97.9	67	87	70.1	1.51	85	200	0
106.4	73	86	74.5	2.05	81	250	0
103	86	100	73	1.18	146	210	0
115	61	93	75	1.22	98	310	0
117	74	79	88	1.13	52	360	0
112	79	95	83	1.05	120	240	0
126	80	112	80	1.3	145	320	0

124	84	102	87	1.2	158	280	0
103	70	91	78	1.36	121	150	0
112	78	72	75	1.02	100	330	0
109	89	99	69	1.36	123	400	0
107	82	93	59	1.23	99	240	0
106	65	54	57	1.08	80	210	0
104	90	90	73	1.41	96	250	0
125	84	72	88	1.05	97	220	0
118	100	100	77	1.1	83	300	0
114.3	72	73	76	1.34	87	180	0
115	70	78	88	1.07	150	320	0
102	72	88	64	1.2	185	160	0

麻黄素mg	苯肾ug	阿托品mg	失血量ml	尿量ml	晶体ml	胶体ml	RBCU	血浆ml
6	0	0	50	200	1000	0	0	0
12	0	0	100	300	1100	0	0	0
30	0	0	200	500	1600	0	0	0
0	0	0	150	400	1100	0	0	0
6	0	0	700	700	1500	1000	0	0
6	600	0	50	200	600	0	0	0
0	0	0	50	600	1100	0	0	0
6	0	0	50	600	1600	0	0	0
12	0	0.3	20	200	1100	0	0	0
6	0	0	50	500	1100	0	0	0
0	0	0	50	100	1100	0	0	0
0	0	0	60	200	1100	500	0	0
6	0	0	100	800	2100	0	0	0
6	40	0	10	700	1100	0	0	0
12	0	0	50	200	1100	0	0	0
0	0	0	50	200	2100	0	0	0
15	0	0	50	400	1500	0	0	0
12	0	0	100	200	2000	0	0	0
12	0	0	50	300	1600	0	0	0
6	0	0	50	400	1500	0	0	0
6	0	0	0	100	1100	0	0	0
12	0	0	30	500	1750	0	0	0
0	0	0	20	200	1600	0	0	0
0	0	0	20	200	1600	0	0	0
6	100	0.2	20	50	1100	0	0	0
12	0	0	20	1000	1000	500	0	0
24	0	0	100	200	1100	0	0	0
6	0	0	100	900	1600	0	0	0
12	0	0	10	700	1600	0	0	0
6	0	0	50	100	1500	0	0	0
6	0	0	20	400	1600	0	0	0
0	0	0	50	600	1500	0	0	0
10	0	0	0	600	2000	0	0	0
16	0	0.3	50	100	1500	0	0	0
0	0	0	30	200	800	0	0	0
6	0	0	30	100	1500	0	0	0
0	0	0	50	150	1100	0	0	0
3	0	0	50	250	1500	0	0	0
0	0	0	0	50	1600	0	0	0
18	0	0	50	300	1500	0	0	0
12	50	0	100	500	1100	500	0	0
0	0	0	50	300	1500	0	0	0
0	0	0	50	200	1500	0	0	0
3	0	0	50	700	2000	0	0	0
6	0	0	150	200	1500	0	0	0
0	0	0	50	300	1600	0	0	0
0	0	0	100	900	2100	0	0	0
0	0	0	50	280	1500	0	0	0

0	0	0	50	400	1500	0	0	0
0	0	0	50	100	1600	0	0	0
0	0	0	50	200	1600	0	0	0
12	0	0	400	200	1000	500	0	0
6	0	0	50	900	1600	0	0	0
6	0	0	50	510	1000	0	0	0
6	0	0	40	79	59	0	0	0
0	0	0	200	200	1000	0	0	0
12	0	0	50	50	1100	0	0	0
0	0	0	50	150	1100	0	0	0
0	0	0	10	200	1100	0	0	0
6	160	0	50	800	1800	0	0	0

术前疼痛评分	RestVAS	R4hVAS	R12hVAS	R24hVAS	R48hVAS	ActionVAS	A4hVAS
0	0	0	5	2	2	1	2
0	0	0	2	2	1	0	2
0	0	2	3	1	1	0	4
0	2	5	4	1	1	2	5
0	1	0	0	1	1	2	0
0	0	3	2	0	3	2	5
0	0	2	2	0	0	0	4
0	0	2	2	2	0	0	3
0	0	2	0	0	2	2	4
0	0	0	2	1	0	0	1
0	0	2	3	4	2	0	4
0	4	0	3	3	0	0	2
0	2	5	5	3	3	0	6
0	0	2	4	3.5	3.5	0	3
0	0	2	1	0	0	0	3
0	0	2	3	0	0	0	3
0	0	0	4	3.5	0	1	3
0	0	0	0	0	0	0	3
0	2	3	3	4	1	2	4
0	0	0	1	2	2	0	0
2	0	0	0	0	0	0	1
0	2	3	3	0	0	2	3
0	0	0	1	1	0	0	2
1	0	1	4	2	0	0	2
0	0	0	1	1	1	0	0
1	3	3	3	1	1	3	3
0	0	2.5	4.5	2	0	0	4.5
0	0	1	2	2	1	1	2
0	0	3	4	2	2	1	2
0	0	0	2	2	0	0	1
0	2	0	0	0	0	3	1
0	3	1	1	2	1	3	2
0	0	0	0	0	0	0	2
0	0	1	1	1	0	0	2
0	0	0	0	0	0	0	2
0	0	2	3	3	2	0	3
0	0	3	2	1	0	0	6
0	1	4	1	1	1	4	5
0	0	0	3	2	2	0	3
0	0	0	4	1	3	0	2
0	0	0	0	0	0	2	2
3	0	2	5	2	1	0	4
0	2	2	3	2	1	2	4
0	0	2	2	2	0	0	2
0	2	1	0	0	0	0	2
0	2	3	5	4	2	3	3
0	2	2	5	1	1	3	3
0	0	2	2	4	2	2	3

0	2	2	4	4	2	4	4
0	2	2	5	2	1	3	3
0	1	1	1	0	1	0	2
0	0	2	5	2	1	1	4
0	0	0	0	0	0	0	0
0	0	2	3	2	0	0	3
0	3	3	4	5	4	5	5
0	0	4	3	3	1	0	6
0	0	3	3	3	2	2	5
0	0	2	3	4	3	2	5
0	0	0	2	1	0	0	2
0	0	0	0	0	0	0	2

A12hVAS	A24hVAS	A48hVAS	PCIA4h	PCIA12h	PCIA24h	PCIA48h	补救镇痛药
4	3	2	3	6	9	10	0
4	3	2	0	1.5	1.5	4.5	0
5	3	2	4	8	9	11.5	0
5	4	4	0	1.5	1.5	4.5	1
2	3	2	0	0	3	3	0
4	2	1	0	4.5	6	9	0
4	5	3	1.5	1.5	3	4.5	1
4	4	2	0	2	4	8	1
2	2	5	0	0	3	4.5	0
4	3	2	0	0	1	2	0
5	5	3	0	6	10	14	0
4	4.5	4	2	3	3	6	0
4	3.5	3	2	4	6	8	0
4	4	3	0	4	8	12	0
2	2	0	4.5	4.5	4.5	9	0
5	3	1	0	1	3	4.5	0
5	5	3	1.5	3	3	3	1
2.5	2	1	4.5	8	9	13.5	0
4	5	2	1.5	1.5	1.5	6	0
3	4	3	0	1.5	2	3	0
3	2	2	0	2	2	3	0
3	3	1.5	1.5	1.5	1.5	4.5	0
2	3	2	1	2	2	3	0
5	3	2	0	1.5	3	6	0
2	2	2	0	0	1.5	3	0
3	2	2	0	0	3	3	0
4.5	4	2	0	1	2	5	0
4	3	3	0	1.5	3	6	0
3	4	3	1.5	4.5	6	9	0
3	3	5	1.5	4.5	7.5	9	0
1	1	1	1	3	4.5	9	1
1	2	1	0	0	1.5	4.5	0
3	3	1	0	2	6	10	1
2	3	2	4.5	9	10.5	16	0
2	3	0	0	3	4.5	7.5	0
4	4	4	4.25	9	11.75	11.75	0
3	1	1	4.5	14	15	15	0
2	2	2	8	16	18	20	0
5	3	3	0	0	4	10	0
5	2	5	0	1.5	1.5	4.5	0
2	2	1	1.5	1.5	7.5	16.5	0
6	6	4	0	1	3	6	0
5	4	2	3	4.5	7.5	7.5	0
2	3	1	0	1.5	7.5	9	1
1	1	0	2	4	4	4	0
5	5	3	0	3	4.5	4.5	0
6	2	3	0	6	12	12	0
3	6	4	1.5	9	13.5	19	0

5	5	3	1.5	8.5	12	12	1
6	3	3	1.5	6	7.5	7.5	0
2	1	2	3	3	3	4.5	0
6	3	3	1	2	6	6	0
2	3	4	3	4.5	6	9	0
5	4	3	0	0	2	5	0
6	5	4	1.5	8	11	13.5	0
6	6	4	1.5	3	3	10.5	0
4	4	3	2	6	16	18	0
5	6	5	1.0625	3.0625	5.0625	17.2325	0
3	4	3	3	9	19.5	22	0
3	3	1	0	1.5	3	6	0

恶心发生率	呕吐发生率	瘙痒发生率	呼吸费力发生率	排气时间	下地时间
0	0	0	0	39.22	72.97
0	0	0	0	48.23	48.23
0	0	0	0	52.87	71.87
0	0	0	0	17.20	46.70
0	0	0	0	26.33	62.33
0	0	0	0	43.92	72.92
0	0	0	0	53.50	17.50
1	1	0	0	25.23	36.73
0	0	0	0	51.95	49.95
1	1	0	0	45.45	41.95
0	0	0	1	77.37	52.37
1	1	0	1	41.87	49.37
0	0	0	0	18.50	47.00
1	1	0	0	40.67	48.08
0	0	0	0	68.17	45.17
0	0	0	0	11.93	40.93
0	0	0	0	48.12	44.12
0	0	0	0	20.47	26.00
1	1	1	1	47.65	71.15
0	0	0	0	29.20	51.20
0	0	0	0	20.67	49.67
0	0	0	0	19.08	48.08
1	1	0	0	38.20	38.20
0	0	0	0	53.00	43.00
0	0	0	0	56.92	45.92
1	1	0	0	23.67	44.33
1	1	0	0	7.88	73.88
0	0	0	0	25.53	44.03
0	0	0	0	34.75	69.75
0	0	0	0	47.08	43.08
0	0	0	0	21.02	43.52
1	1	0	0	42.22	67.05
0	0	0	0	11.17	70.17
0	0	0	0	67.58	20.58
0	0	0	0	18.75	40.75
0	0	0	0	28.08	71.58
1	1	0	0	33.50	42.50
1	1	0	0	40.33	47.83
1	1	0	0	16.35	70.85
0	0	0	0	12.88	13.38
0	0	0	1	74.83	63.83
1	1	0	0	43.23	65.23
0	0	0	0	51.67	70.67
0	0	0	0	33.40	62.90
0	0	0	0	43.00	66.00
0	0	0	0	39.83	48.83
1	1	0	0	15.53	48.53
0	0	0	0	50.87	74.87

1	1	0	0	17.12	68.12
0	0	0	0	24.82	43.32
0	0	0	0	28.32	22.82
0	0	0	0	38.90	37.40
0	0	0	0	28.08	43.08
0	0	0	1	17.45	66.45
1	1	0	0	46.95	46.95
1	1	0	0	46.00	70.50
0	0	0	0	42.75	27.75
0	0	0	1	71.65	68.32
0	0	0	0	29.00	26.00
1	1	0	0	36.08	66.08

拔尿管实际时间	评估满意度	苏醒满意度	镇痛满意度	恶心呕吐治疗满意度	麻醉满意度
72.97	5	5	4	3	5
47.23	3	4	5	3	5
71.87	4	5	5	5	5
44.70	4	4	4	4	4
62.33	5	5	5	5	5
69.92	5	5	5	5	5
42.50	4	4	4	4	4
37.23	4	4	4	4	4
52.95	4	4	5	5	5
54.45	5	5	4	4	5
77.37	4	5	5	3	5
47.87	4	4	4	4	5
65.50	5	5	5	5	5
77.42	5	5	5	5	5
75.17	5	5	4	5	5
20.93	5	5	5	5	5
44.12	5	5	5	3	5
43.00	4	4	4	4	4
70.15	5	5	5	2	5
51.20	5	5	5	5	5
64.67	5	5	5	3	5
58.08	5	5	5	5	5
40.20	5	4	5	4	5
48.00	5	5	4	4	5
53.42	4	4	4	3	4
42.67	5	5	5	5	5
73.88	5	5	5	4	5
62.53	4	4	4	3	5
85.75	5	5	5	5	5
52.08	5	5	5	5	5
43.02	4	5	5	3	5
72.05	4	4	3	5	4
47.67	4	5	4	4	9
45.58	5	5	4	5	5
43.75	5	5	5	5	5
71.08	4	5	4	5	5
74.50	4	4	4	3	4
20.17	5	5	4	5	5
68.18	4	4	5	4	5
19.38	5	5	5	5	5
86.83	4	4	4	3	5
71.23	5	5	4	5	5
71.67	5	5	5	5	5
62.40	5	4	5	5	5
69.00	4	4	4	4	4
39.83	5	5	5	5	5
115.03	5	5	5	5	5
69.87	4	4	4	4	4

67.12	4	4	4	4	4
47.32	4	5	5	4	5
64.32	5	5	4	4	5
37.40	4	5	4	5	5
49.08	5	5	5	5	5
21.45	4	3	4	4	4
38.95	5	5	4	4	5
68.50	4	4	3	4	4
70.75	4	3	4	3	4
68.32	5	5	5	5	5
52.00	5	5	5	5	5
65.08	5	5	5	5	5

QoR3d	QoR5d	住院时间
109	129	7
127	141	6
106	137	5
139	148	4
112	139	10
144	148	5
84	88	5
142	150	3
119	144	8
112	139	6
128	150	6
118	132	7
106	128	6
92	120	6
117	128	5
145	148	6
133	144	4
140	144	5
117	139	7
109	119	7
134	141	4
131	147	3
146	149	3
141	148	7
139	141	7
133	144	6
139	150	7
115	132	7
117	138	6
128	143	5
135	142	3
134	141	6
119	145	7
144	149	4
150	150	4
115	135	6
120	133	5
96	137	7
137	144	7
126	150	5
130	150	7
143	150	5
144	150	6
129	141	7
120	146	7
132	120	7
120	112	6
105	133	5

124	136	5
131	137	7
118	147	7
122	128	7
139	150	5
141	148	7
140	150	5
132	120	11
117	134	6
120	132	5
137	131	5
115	134	5
