# Supplementary material for: Comparison of the Postoperative Analgesic Effects between Ultrasound-Guided Transmuscular Quadratus Lumborum Block and Thoracic Paravertebral Block in Laparoscopic Partial Nephrectomy Patients: A Randomized, Controlled, and Noninferiority Study
Source: Pain Res Manag. 2023 Feb 20;2023:8652596. doi: 10.1155/2023/8652596 (PMC9988391; doi:10.1155/2023/8652596)
Supplement: Supplementary Materials — Supplement 1. CONSORT 2010 Checklist. Supplement 2. CONSORT Extension for Noninferiority and Equivalence Trials Checklist 2006. Supplement 3. Abstracts Checklist. The raw data is provided in Supplementary Materials. [file 8652596.f1.zip › Supplement 3-Abstracts Checklist.doc]

**Items to include when reporting a randomized trial in a journal or conference abstract**

| **Item** | **Description** | **Reported on line number** |
| --- | --- | --- |
| Title | Identification of the study as randomized | Page 1, line 3. |
| Authors * | Contact details for the corresponding author | Page 1, line 4-5, 13-17. |
| Trial design | Description of the trial design (e.g. parallel, cluster, non-inferiority) | Page 2, line 2. |
| Methods |  |  |
| Participants | Eligibility criteria for participants and the settings where the data were collected | Page 2, line 5-6. |
| Interventions | Interventions intended for each group | Page 2, line 7-9. |
| Objective | Specific objective or hypothesis | Page 2, line 2-4, 10-13 |
| Outcome | Clearly defined primary outcome for this report | Page 2, line 10-11. |
| Randomization | How participants were allocated to interventions | Page 2, line 6-7. |
| Blinding (masking) | Whether or not participants, care givers, and those assessing the outcomes were blinded to group assignment | Page 2, line 9-10. |
| Results |  |  |
| Numbers randomized | Number of participants randomized to each group | Page 2, line 5. |
| Recruitment | Trial status | Page 2, line 14. |
| Numbers analysed | Number of participants analysed in each group | Page 2, line 14. |
| Outcome | For the primary outcome, a result for each group and the estimated effect size and its precision | Page 2, line 14-17. |
| Harms | Important adverse events or side effects | Page 2, line 21. |
| Conclusions | General interpretation of the results | Page 3, line 1-2. |
| Trial registration | Registration number and name of trial register | Page 3, line 3. |
| Funding | Source of funding | Page 3, line 4-5. |

**this item is specific to conference abstracts*
